# Deltamethrin resistance in *Aedes aegypti* results in treatment failure in Merida, Mexico

**DOI:** 10.1371/journal.pntd.0005656

**Published:** 2017-06-12

**Authors:** Gonzalo M. Vazquez-Prokopec, Anuar Medina-Barreiro, Azael Che-Mendoza, Felipe Dzul-Manzanilla, Fabian Correa-Morales, Guillermo Guillermo-May, Wilbert Bibiano-Marín, Valentín Uc-Puc, Eduardo Geded-Moreno, José Vadillo-Sánchez, Jorge Palacio-Vargas, Scott A. Ritchie, Audrey Lenhart, Pablo Manrique-Saide

**Affiliations:** 1 Department of Environmental Sciences, Emory University, Atlanta, Georgia, United States of America; 2 Unidad Colaborativa de Bioensayos Entomológicos, Campus de Ciencias Biológicas y Agropecuarias, Universidad Autónoma de Yucatán, Merida, Yucatan, Mexico; 3 Centro Nacional de Programas Preventivos y Control de Enfermedades (CENAPRECE) Secretaría de Salud Mexico, Ciudad de Mexico, Mexico; 4 Secretaria de Salud de Yucatan, Merida, Yucatan, Mexico; 5 College of Public Health, Medical and Veterinary Sciences, James Cook University, Cairns, Queensland, Australia; 6 Australian Institute of Tropical Health and Medicine, James Cook University, Cairns, Queensland, Australia; 7 Centers for Disease Control and Prevention, Atlanta, Georgia, United States of America; North Carolina State University, UNITED STATES

## Abstract

The operational impact of deltamethrin resistance on the efficacy of indoor insecticide applications to control *Aedes aegypti* was evaluated in Merida, Mexico. A randomized controlled trial quantified the efficacy of indoor residual spraying (IRS) against adult *Ae*. *aegypti* in houses treated with either deltamethrin (to which local *Ae*. *aegypti* expressed a high degree of resistance) or bendiocarb (to which local *Ae*. *aegypti* were fully susceptible) as compared to untreated control houses. All adult *Ae*. *aegypti* infestation indices during 3 months post-spraying were significantly lower in houses treated with bendiocarb compared to untreated houses (odds ratio <0.75; incidence rate ratio < 0.65) whereas no statistically significant difference was detected between the untreated and the deltamethrin-treated houses. On average, bendiocarb spraying reduced *Ae*. *aegypti* abundance by 60% during a 3-month period. Results demonstrate that vector control efficacy can be significantly compromised when the insecticide resistance status of *Ae*. *aegypti* populations is not taken into consideration.

## Author summary

Insecticide resistance has emerged as a worrisome outlook for the implementation of insecticide-only approaches. Particularly for *Aedes aegypti*, it is generally argued that this rapid rise of insecticide resistance may compromise the effectiveness of control programs. Yet, empirical evidence of such negative operational impact is lacking. This article provides quantitative evidence of the impact of insecticide resistance, primarily to the pyrethroid insecticide deltamethrin, on the entomological effectiveness of interventions against *Aedes aegypti* mosquitoes. Within an area of high resistance to pyrethroids, performing high-quality insecticide applications using deltamethrin had no entomological impact in comparison to the application of an insecticide to which *Ae*. *aegypti* was susceptible. Deltamethrin did not protect against resistant *Ae*. *aegypti*, whereas the application of bendiocarb led to an average efficacy of 60% during a 3-month period. This study provides strong evidence of the dramatic operational treatment failure that can occur when the background insecticide resistance level of *Ae*. *aegypti* populations is not taken into consideration when performing vector control.

## Introduction

The expanding geographic range of *Aedes aegypti* and increased human mobility have primed the world for increased transmission of *Aedes-*borne diseases [1, 2]. Zika is rapidly propagating and causing severe congenital complications in the Americas [3], yellow fever has recently re-emerged in urban Africa [4], Mayaro virus is gaining prevalence [5], and dengue viruses continue to be the most prevalent mosquito-borne arboviruses worldwide [6]. With the exception of yellow fever, which can be prevented with a vaccine, the containment of the remaining *Aedes*-borne viruses depends almost exclusively on vector control and community mobilization [7, 8]. Unfortunately, *Aedes*-control programs today are challenged by limited budgets and the social-environmental complexities of contemporary urban areas. Given that existing vector control methods are context-dependent and can vary greatly in effectiveness [7, 9], and in light of the increasing threat posed by *Aedes*-borne diseases, there is an urgent need to determine which vector control tools and strategies provide the greatest impact [7].

One of the key challenges with insecticide-based interventions is that they inherently select for insecticide resistance [10]. In multiple countries, resistance to pyrethroid insecticides has been reported in *Ae*. *aegypti*, likely resulting from the widespread reliance of vector control programs on pyrethroid insecticides for over two decades [11]. The most widely reported mechanisms of pyrethroid resistance in *Ae*. *aegypti* are increased detoxification due to P450-monooxygenases and mutations in the voltage-gated sodium channel genes [11]. In Mexico, the widespread use of permethrin over a 10-year period led to the rapid emergence and spread of a mutation in codon 1016 of the gene encoding the voltage-gated sodium channel that results in an isoleucine substitution for valine [12]. An additional mutation in codon 1534 resulting in a cysteine substitution for phenylalanine has also been detected at high frequency throughout Mexico, and is thought to have evolved to compensate for fitness costs incurred by 1016I [13]. Both 1016I and 1534C kdr mutations are now widespread throughout Mexico, and have reached fixation or near-fixation in many places in only a few short years. This example of rapid evolution has been documented elsewhere [11], and represents a worrisome outlook for the reliance on insecticide-only approaches. Particularly for *Ae*. *aegypti*, it is generally argued that the rapid rise of insecticide resistance may compromise the effectiveness of control programs [10, 14], hindering the ability to control pathogen transmission, yet empirical evidence of such impact is lacking. This lack of evidence is at least partially due to the variability in the effectiveness of interventions [9], which can confound the accuracy of measurements of both the entomological and epidemiological impacts of resistance.

Indoor residual spraying (IRS), when targeted to *Ae*. *aegypti* resting locations, can provide a significant protective effect against dengue transmission [15, 16]. In addition to being one of the only vector control interventions clearly and significantly linked to a reduction in disease transmission, this method also has potential for the control of pyrethroid-resistant *Ae*. *aegypti*, as non-pyrethroid insecticides are available for residual application. As such, a randomized controlled trial (RCT) was conducted in the city of Merida, Mexico, with two objectives: 1) to quantify the efficacy of IRS in controlling *Ae*. *aegypti*, and 2) to evaluate the operational impact of pyrethroid resistance by comparing the efficacy of interventions using an insecticide to which the *Ae*. *aegypti* population presented a high frequency of resistance as compared to an insecticide to which the *Ae*. *aegypti* population was susceptible.

## Materials and methods

### Study area

**Fig 1:**
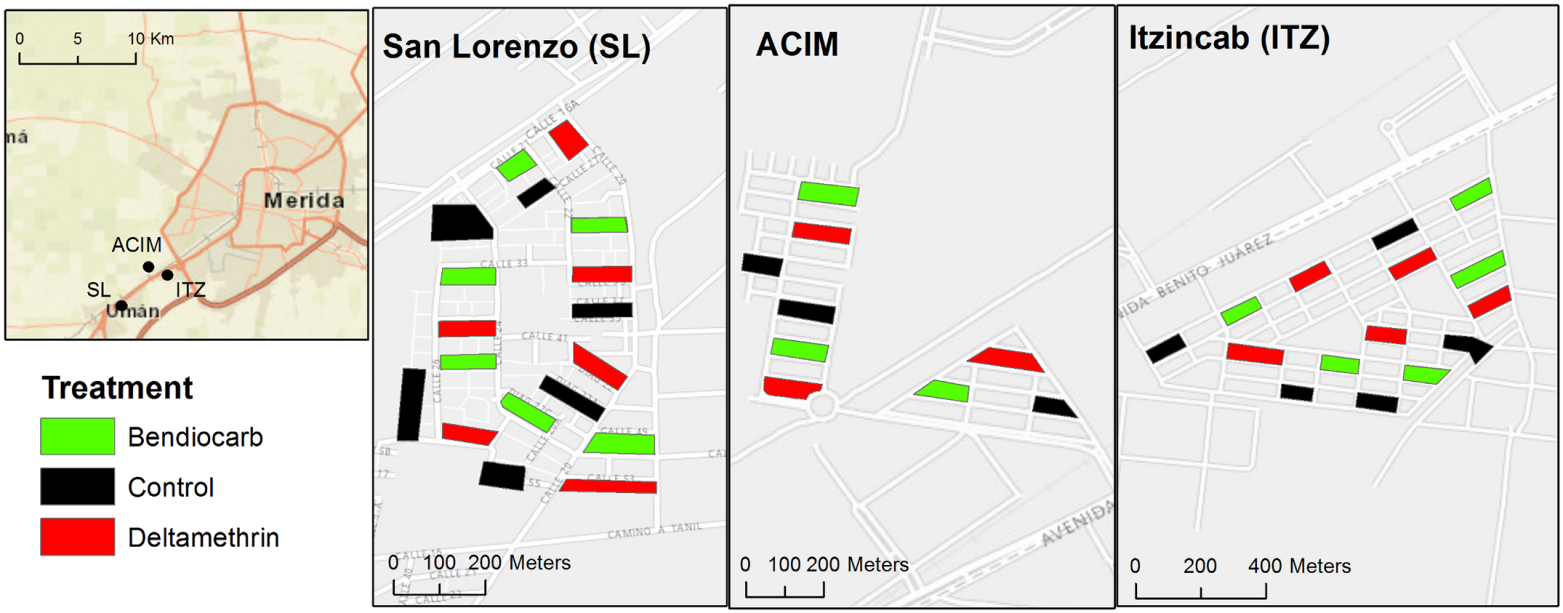
Map of the location of the three Merida suburbs (inset) and distribution of treatment and control blocks within each.

This study was conducted in the state of Yucatán in southern Mexico in three suburbs (San Lorenzo, Acim, Itzincab) of Merida (population ~1 million), the state’s capital (Fig 1). The three suburbs were small, densely populated ‘fraccionamientos’ (neighborhoods) connected to Merida by a single road and similar in housing size and design (e.g., one story, brick-and-mortar homes with typically two bedrooms, one living room, one TV room, a bathroom and a kitchen), characteristic of high-density low-income housing in the region. Merida is located in a subtropical environment with mean temperatures ranging from 29°C in December to 34°C in July. The rainy season occurs from May to October and overlaps with the peak dengue transmission season between July and November, although cases occur year-round [17]. Dengue virus is widely distributed throughout the Yucatan peninsula, and the vector control strategies used by local authorities at the time of this study included ultra-low volume (ULV) spraying with the organophosphate insecticides chlorpyrifos and malathion and indoor space spraying with pyrethroids (deltamethrin) and organophosphates (malathion) for adult *Ae*. *aegypti* control. Recent published reports from the region categorized the populations of *Ae*. *aegypti* as resistant to type I and II pyrethroids (including deltamethrin) and completely susceptible to carbamates (including bendiocarb) [18]. Mutations had previously been detected at both loci on the voltage-gated sodium channel gene, resulting in the presence of both 1016I and 1534C [18, 19].

### Study design

To test the hypothesis that insecticide resistance can impact the efficacy of insecticide-based interventions, a RCT was designed with three study arms: blocks where IRS (see below) was conducted using deltamethrin (WP, 5 g a.i. diluted in 7.5 L of water), blocks where modified IRS was conducted with bendiocarb (WP, 125 g sachet diluted in 7.5 L of water) and untreated control blocks. WHO recommended doses were used for each insecticide (0.025 g active ingredient [a.i.]/m^2^ for deltamethrin and 0.375 g a.i./m^2^ for bendiocarb) which were applied using standard manual compression sprayers (Hudson 93793 X-pert, Chicago, IL) with flat nozzle fitted with flow control valves (CFV red, model CFV.R11/16SYV.ST, with operating pressure 1.5Bar/21 psi, Flow rate 550 ml/min, CFValve, Gate LLC, Sebastian, FL).

Under strong spatial heterogeneity, as it is observed for *Ae*. *aegypti* [20], randomly allocating treatments over space can lead to marked variability in the distribution of treatment and control blocks. This challenge can be addressed with a randomized-block design by allocating treatments to smaller geographic units (blocks) nested within larger ones [21]. In this study, 14 clusters containing three city blocks each were generated as follows: 1) 42 city blocks were randomly selected using a geographic information system (ArcGIS 10.1, Redlands, CA), restricting the selection to blocks that were not contiguous (one city block was left as a buffer to reduce confounding between treatments due to mosquito dispersal); 2) the 42 city blocks were then used to generate ‘clusters’ of 3 blocks (by selecting blocks that were closest to each other based on the distance between their centroids); 3) each of the 3 blocks within the cluster was randomly allocated to one of the study arms (control, deltamethrin, bendiocarb)(Fig 1).

Two to three weeks prior to implementation of an intervention in a block, written informed consent from the household owner was obtained. Blocks with enrollment <60% were replaced by newly selected blocks, as previous studies showed that the epidemiologic effectiveness of IRS is significantly lower below such threshold [15]. One week prior to the intervention, a baseline entomologic survey using ovitraps [22], Prokopack aspirators to collect adult mosquitoes for 10 minutes per house [23] and pupal surveys [24] was systematically conducted at 10 randomly selected houses per block to determine pre-treatment vector abundance and insecticide resistance prevalence. Susceptibility to deltamethrin and bendiocarb was assessed with CDC bottle bioassays on adult mosquitoes emerging from the eggs hatched from ovitraps placed in each cluster. Resistance intensity was measured by exposing mosquitoes to multiples of the diagnostic dose (2x, 5x and 10x) [25].

Spraying occurred from October 31 to November 22, 2015. Three teams of 2 research team staff who had been trained in the application of insecticides adapted the standardized IRS protocols used for malaria vector control [26] to the urban control of *Ae*. *aegypti*. While the technical parameters for spraying (distance from wand to wall, speed, pressure, etc.) were kept as used in classic IRS, some modifications were introduced to maximize application of insecticides to specific *Ae*. *aegypti* resting sites [27] and increase acceptability by house owners [28]: 1. No personal belongings were taken outside of the home and furniture was not moved away from the walls; only exposed walls were sprayed; 2. Picture frames and other belongings hanging on the walls were kept unless the owner decided to remove them; 3. Kitchens were not sprayed to minimize the risk of food contamination and because evidence from exhaustive collections shows that *Ae*. *aegypti* is rarely found resting there [27].

In Iquitos, Peru, IRS with deltamethrin induced mortalities higher than 80% in susceptible *Ae*. *aegypti* strains up to eight weeks post spraying but mortality was reduced to 55% at 16 weeks post-spraying [29]. Thus, we designed our post-intervention (PI) entomological evaluations to occur at 15 days, 1 month, 2 months and 3 months post-spraying. On each survey date, 10 randomly selected houses per block were surveyed using the same methodology as at baseline. All collected adult mosquitoes were transported to the laboratory (Universidad Autonoma de Yucatan, Merida) in styrofoam coolers for further processing. Once in the lab, mosquitoes were separated by species, sex and (for females) sorted by the level of engorgement as recently bloodfed or non-bloodfed. Mosquitoes were individually stored in labeled vials containing RNALater (Invitrogen, Carlsbad, CA) and kept at -80°C for future processing. Cone bioassays [30] using a susceptible laboratory strain of *Ae*. *aegypti* (New Orleans) were performed monthly in a random selection of 3 houses per treatment arm to determine the residual effect of the insecticide treatments. A sample of 100 *Ae*. *aegypti* females collected in the field was randomly selected at baseline to quantify the initial frequency of the most common knock-down resistance mutations. Genomic DNA was extracted from a leg or other body part from each individual mosquito and kdr allele-specific assays were be performed using real-time PCR. DNA extractions were performed by mixing the mosquito body part with a 50ul solution containing 5ul of Taq 10X buffer (containing 500mM KCl, 100mM tris HCl, 15mM MgCl2, and 1% Triton X-100) and 45ul of sterile ddH2O and heating in a thermocycler (Eppendorf Mastercycler pro, Hamburg, Germany) at 95°C for 15 minutes. The 1016I allele was be detected using the methodology described by Saavedra-Rodriguez *et al*. [31] and the 1534C allele by the methodology described by Yanola *et al*. [32].

### Ethics statement

All study protocols were approved by Emory University Institutional Review Board (IRB00082848) as well as the ethics board at the State of Yucatan. Written informed consent was obtained from the household owner was obtained and houses who did not consent to the intervention were noted and not sprayed or visited in post-intervention entomological surveys.

### Statistical analyses

The following *Ae*. *aegypti* adult indices were calculated for each sampling date and compared between treatments and over time: presence (binomial variable) and abundance (count variable) of adults, females and bloodfed females per house. Mean values during all sampling periods were compared using generalized linear mixed effects models (GLMM) nested at the cluster (level 1) and city-block (level 2) levels. Link functions for GLMMs were binomial for presence indices and Poisson for abundance indices. Models were used to calculate odds ratios (OR, for mosquito presence/absence) and incidence rate ratios (IRR, for mosquito abundance) using control houses as the unit of comparison. We calculated the operational efficacy of the intervention as *E* = (1 − *IRR*) × 100. This measure, ranging between 0 and 1, describes the percent reduction of mosquito abundance in treated houses with respect to the control. All models were run with the software package lme4 [33] within the software platform R (https://www.r-project.org/). Data has been made available from the Dryad Digital Repository: http://dx.doi.org/10.5061/dryad.1b070 [34].

## Results

**Fig 2:**
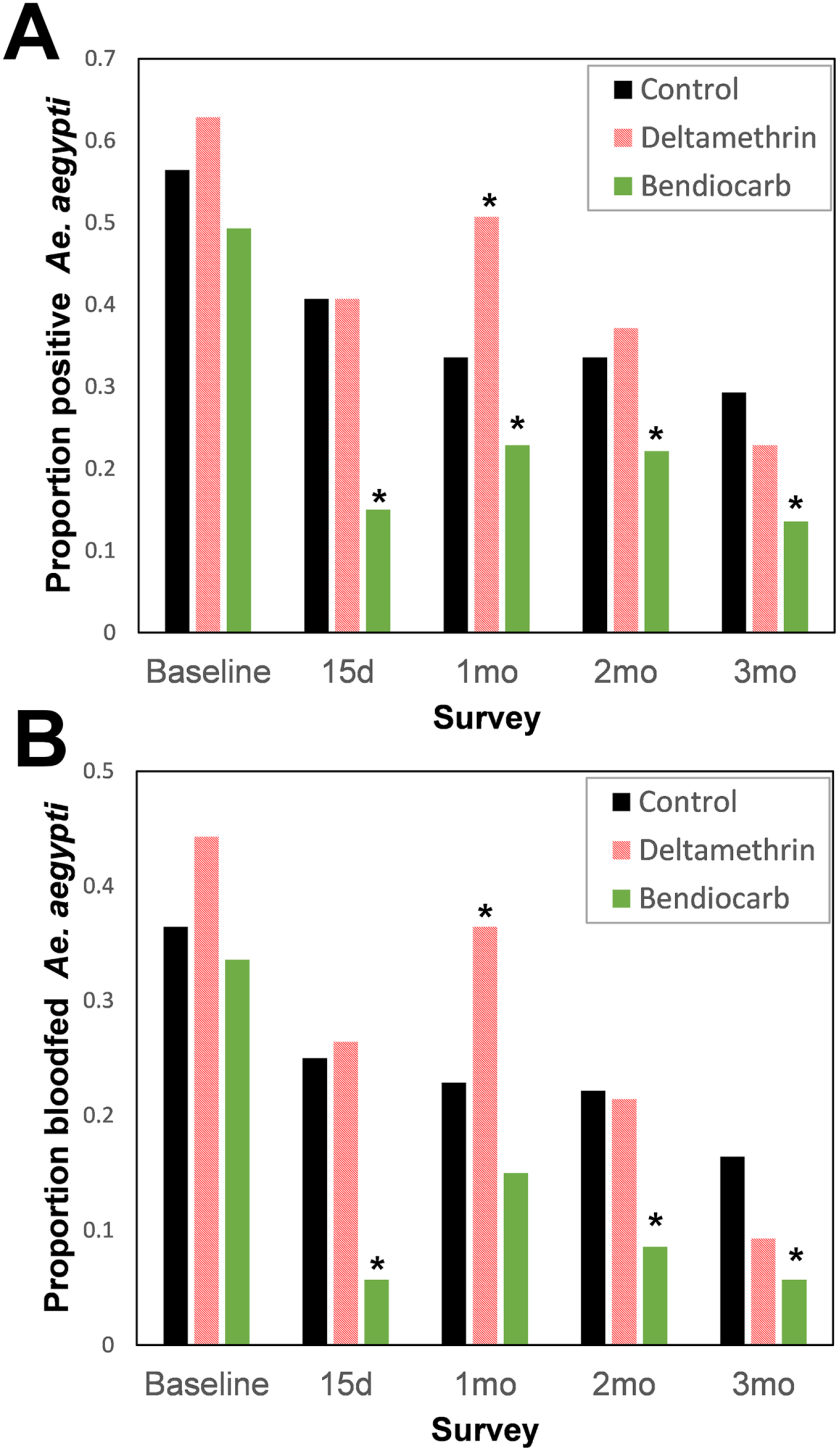
House positivity (proportion of *Ae*. *aegypti* infested houses) by treatment and survey date. Panel (A) shows positivity for adult *Ae*. *aegypti* and panel (B) positivity for bloodfed female *Ae*. *aegypti*. Asterisks (*) indicate statistically significant (P<0.05) difference between each treatment and the control, after a mixed-effects logistic regression model (Table 1).

A total of 2,100 *Ae*. *aegypti*, 1,309 *Aedes taeniorhynchus*, and 1,228 *Culex quinquefasciatus* were collected throughout the trial (140 houses per arm per sampling date, or 420 houses per date, for a total of 1,680 houses in the entire period), of which 39.8%, 81.2% and 10.4% were collected in the baseline survey, respectively. The proportion of houses infested with *Ae*. *aegypti* at baseline ranged between 0.49–0.62; the variability between treatment arms was not statistically significant (generalized linear mixed model, GLMM, |z| < 1.19; *P*>0.234; Fig 2A). The proportion of houses infested with bloodfed females (a more precise index, as bloodfed females have a higher chance of contacting insecticides due to their need to rest immediately after a bloodmeal) at baseline ranged between 0.33–0.44 and did not differ statistically between treatment arms (GLMM, |z| < 1.39; *P*>0.16; Fig 2B). Both indices of adult presence were dramatically reduced post-intervention (Fig 2), with houses treated with bendiocarb being consistently less infested (range, 14–23% for adults and 5–15% for bloodfeds) than houses treated with deltamethrin (range, 23–51% for adults and 9–36% for bloodfeds) or control houses (range, 29–41% for adults and 16–25% for bloodfeds).

Residual spraying with deltamethrin did not produce any measurable impact on any of the *Ae*. *aegypti* presence indices (Table 1). Surprisingly, the odds of finding *Ae*. *aegypti* was significantly higher in clusters treated with deltamethrin compared to unsprayed controls at one month PI (OR > 1.95, *P* < 0.05). Conversely, residual spraying with bendiocarb reduced the positivity of houses, with most adult indices being significantly lower in treated clusters compared to untreated controls (Table 1). Throughout the three month follow-up, houses sprayed with bendiocarb were 0.25–0.58 less likely to have *Ae*. *aegypti* comparison to unsprayed controls. Such protective effect was more marked when looking at female *Ae*. *aegypti* (0.23–0.61) and bloodfed *Ae*. *aegypti* (0.18–0.32)(Table 1).

**Table 1:** Odds ratios (OR) estimated from a mixed-effects logistic regression model evaluating impact of treatment (deltamethrin vs control and bendiocarb vs control) on each adult entomologic metric. The model included city block (where individual observations are nested) and cluster (grouping of 3 treatments) as random intercepts. ORs were calculated by considering control blocks (i.e., unsprayed) as comparison. Numbers in **bold** show statistically significant (P<0.05) difference between the treatment and the control.

Metric	Survey	Deltamethrin			Bendiocarb		
		Coefficient	Lower	Upper	Coefficient	Lower	Upper
**Adult mosquito presence (any species)**	Baseline (pre-spraying)	1.16	0.04	44.91	0.96	0.03	27.56
	15days	0.86	0.53	1.39	**0.26**	0.15	0.44
	1month	2.25	0.53	1.39	**0.75**	0.15	0.44
	2months	1.22	0.76	1.97	1.03	0.64	1.65
	3months	0.79	0.48	1.27	**0.55**	0.33	0.90
**Presence of *Aedes aegypti***	Baseline (pre-spraying)	1.31	0.81	2.17	0.75	0.46	1.20
	15days	1.00	0.61	1.64	**0.25**	0.13	0.44
	1month	**2.06**	1.27	3.43	**0.58**	0.34	0.99
	2months	1.17	0.71	1.93	**0.56**	0.32	0.95
	3months	0.71	0.41	1.22	**0.37**	0.19	0.67
**Presence of *Aedes aegypti* females**	Baseline (pre-spraying)	1.43	0.88	2.35	0.68	0.41	1.10
	15days	1.00	0.61	1.64	**0.23**	0.13	0.44
	1month	**2.11**	1.27	3.37	**0.61**	0.34	0.99
	2months	1.17	0.72	1.92	**0.56**	0.33	0.95
	3months	0.71	0.41	1.22	**0.37**	0.19	0.67
**Presence of bloodfed *Aedes aegypti* females**	Baseline (pre-spraying)	1.39	0.86	2.29	0.88	0.53	1.44
	15days	1.08	0.63	1.85	**0.18**	0.08	0.39
	1month	**1.95**	1.16	3.32	0.59	0.32	1.09
	2months	0.96	0.54	1.70	**0.32**	0.15	0.65
	3months	0.51	0.24	1.05	**0.30**	0.12	0.68

**Fig 3:**
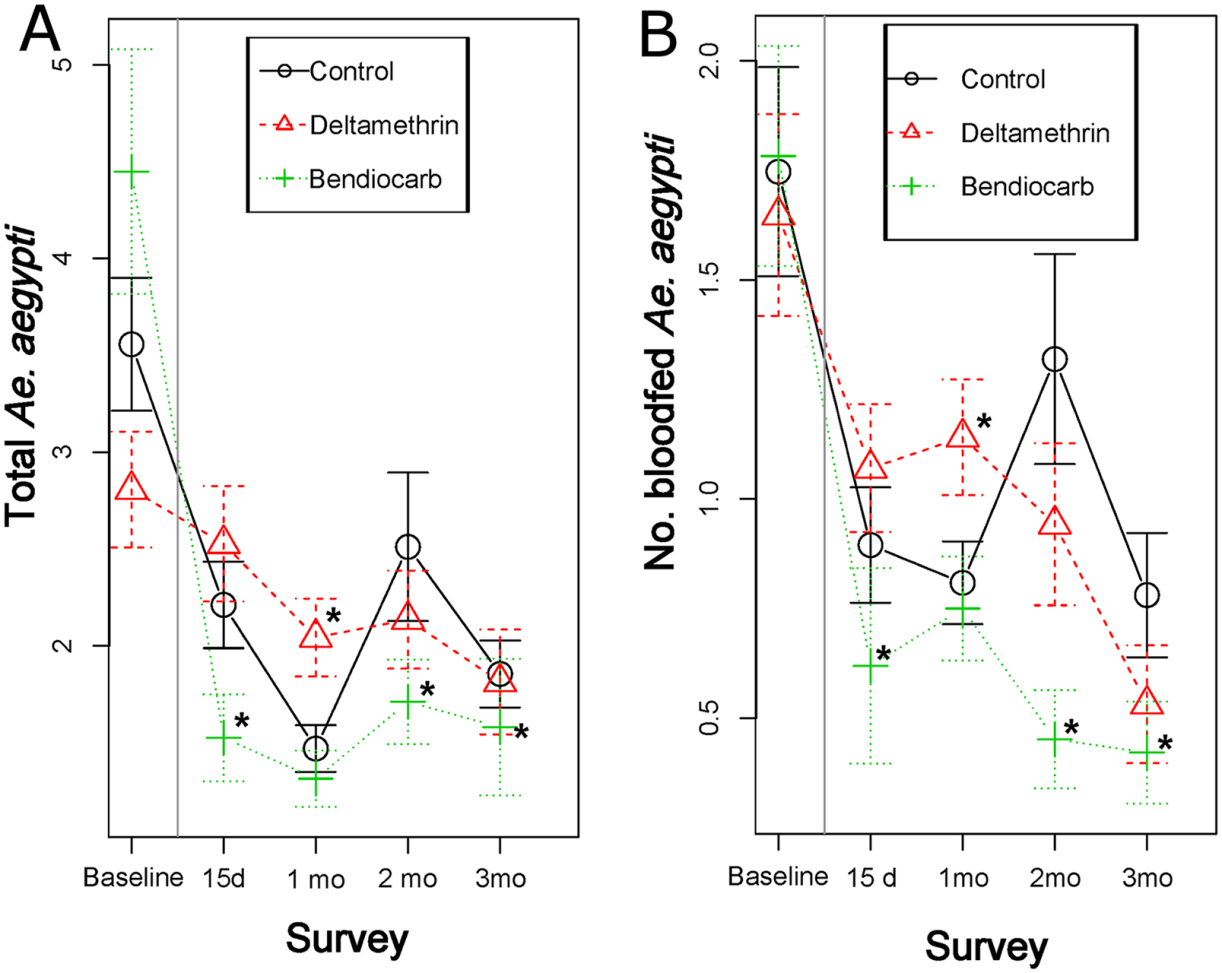
Average (±SE) number of *Ae*. *aegypti* collected per survey date and by treatment. Panel (A) shows positivity for adult *Ae*. *aegypti* and panel (B) positivity for bloodfed female *Ae*. *aegypti*. Vertical gray line indicates the timing of the intervention. Asterisks (*) indicate statistically significant (P<0.05) difference between each treatment and the control, mixed-effects Poisson regression model (Table 2).

At baseline, *Ae*. *aegypti* adult abundance averaged 2.8–4.5 per house and bloodfed *Ae*. *aegypti* 1.6–1.7 per house (Fig 3), with differences between treatment and control clusters lacking statistical significance (Table 2). Spraying with deltamethrin did not produce any measurable impact on the abundance of *Ae*. *aegypti* for all indices evaluated, with the incidence rate ratio (IRR) not differing statistically between deltamethrin vs control clusters (Table 2). However, bendiocarb spraying led to a significant reduction in *Ae*. *aegypti* abundance; IRRs for all adult indices were significantly lower in bendiocarb-sprayed houses compared to untreated controls (Table 2). Based on the IRR values from Table 2, the average efficacy of IRS application of bendiocarb for *Ae*. *aegypti* total abundance (males and females) was 60% during a 3 month period (E_15d_ = 77%, E_1mo_ = 43%, E_2mo_ = 52% and E_3mo_ = 67%). When considering female *Ae*. *aegypti* only, efficacy remained the same (overall, 60%; E_15d_ = 74%, E_1mo_ = 35%, E_2mo_ = 68% and E_3mo_ = 64%).

**Table 2:** Incidence rate ratios (IRR) estimated from a mixed-effects Poisson regression model evaluating effect of treatment (deltamethrin vs control and bendiocarb vs control) on each adult entomologic metric. The model included city block (where individual observations are nested) and cluster (grouping of 3 treatments) as random intercepts. IRRs were calculated by considering control blocks as comparison. Numbers in **bold** show statistically significant (P<0.05) difference between the treatment and the control.

Metric	Survey	Deltamethrin			Bendiocarb		
	Adult abundance	Coefficient	Lower	Upper	Coefficient	Lower	Upper
**No. of adult mosquitoes (any species)**	Baseline (pre-spraying)	0.78	0.61	1.16	1.74	0.78	1.46
	15days	0.73	0.49	1.08	**0.23**	0.14	0.36
	1month	**1.73**	1.21	2.49	0.80	0.55	1.17
	2months	1.10	0.74	1.65	0.80	0.53	1.22
	3months	0.86	0.51	1.44	**0.57**	0.33	0.97
**No. of *Aedes aegypti***	Baseline (pre-spraying)	0.99	0.67	1.46	0.89	0.60	1.32
	15days	1.07	0.66	1.72	**0.23**	0.13	0.41
	1month	**2.21**	1.42	3.50	**0.57**	0.34	0.94
	2months	1.07	0.64	1.78	**0.48**	0.27	0.83
	3months	0.66	0.35	1.22	**0.33**	0.16	0.64
**No. of *Aedes aegypti* females**	Baseline (pre-spraying)	1.05	0.72	1.56	0.82	0.55	1.21
	15days	1.07	0.66	1.75	**0.26**	0.13	0.48
	1month	**2.18**	1.41	3.44	0.65	0.37	1.11
	2months	0.94	0.52	1.71	**0.32**	0.16	0.64
	3months	0.72	0.29	1.80	**0.36**	0.13	0.96
**No. of bloodfed *Aedes aegypti* females**	Baseline (pre-spraying)	1.18	0.72	1.94	0.87	0.52	1.44
	15days	1.19	0.66	2.14	**0.23**	0.10	0.49
	1month	**2.08**	1.31	3.34	0.62	0.35	1.10
	2months	0.86	0.43	1.71	**0.27**	0.11	0.59
	3months	0.52	0.20	1.26	**0.27**	0.09	0.75

**Fig 4:**
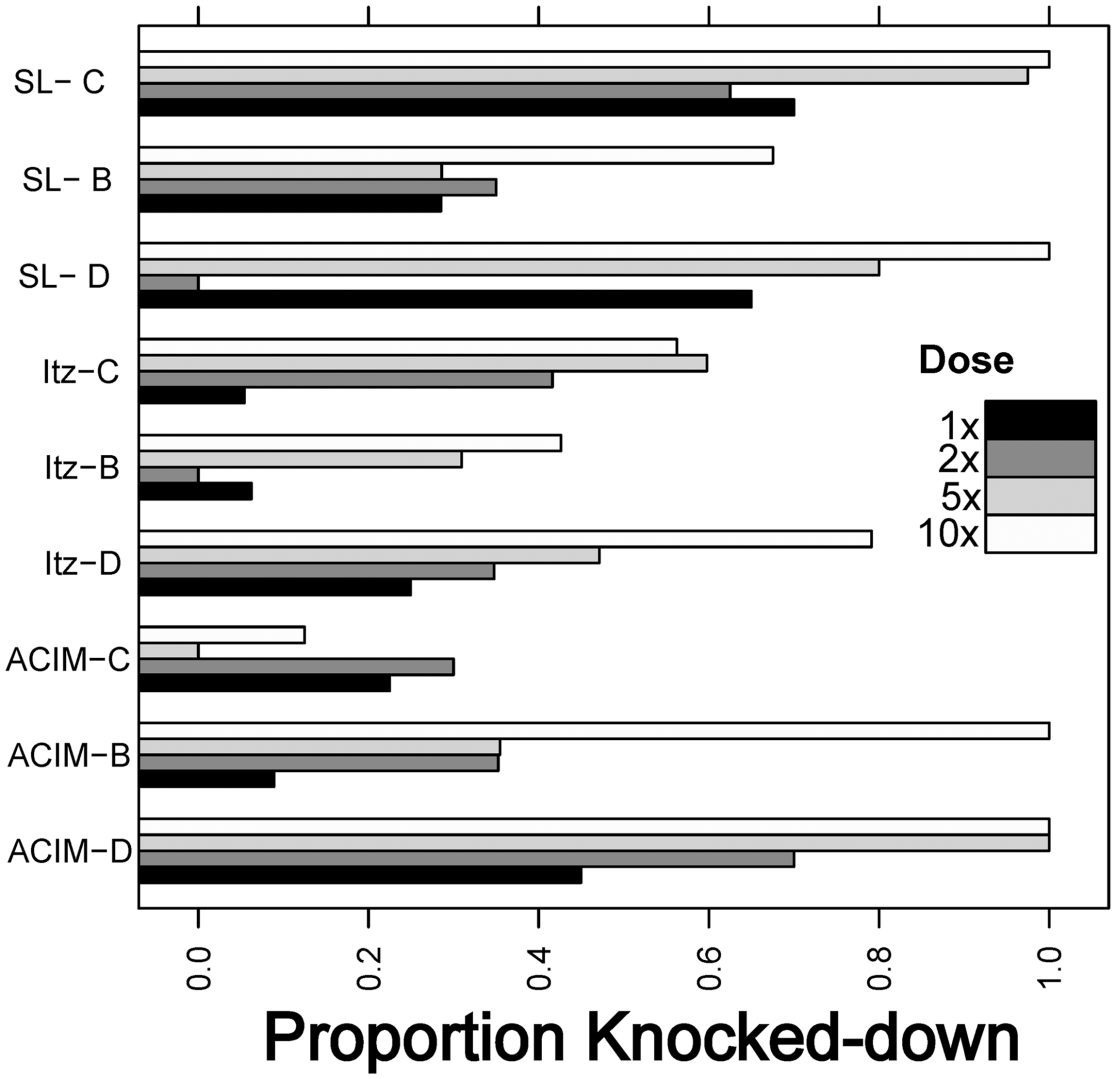
Results from intensity bottle bioassays evaluating the susceptibility of local *Ae*. *aegypti* populations to deltamethrin, defined as knock-down after 30 minutes of exposure to the the diagnostic dose (1x) and at twice, five and ten times the diagnostic dose. Each letter in the Y axis indicates a locality (SL = San Lorenzo, Itz = Itzincab, ACIM = Acim) and treatment (C = control, B = bendiocarb, D = deltamethrin).

**Fig 5:**
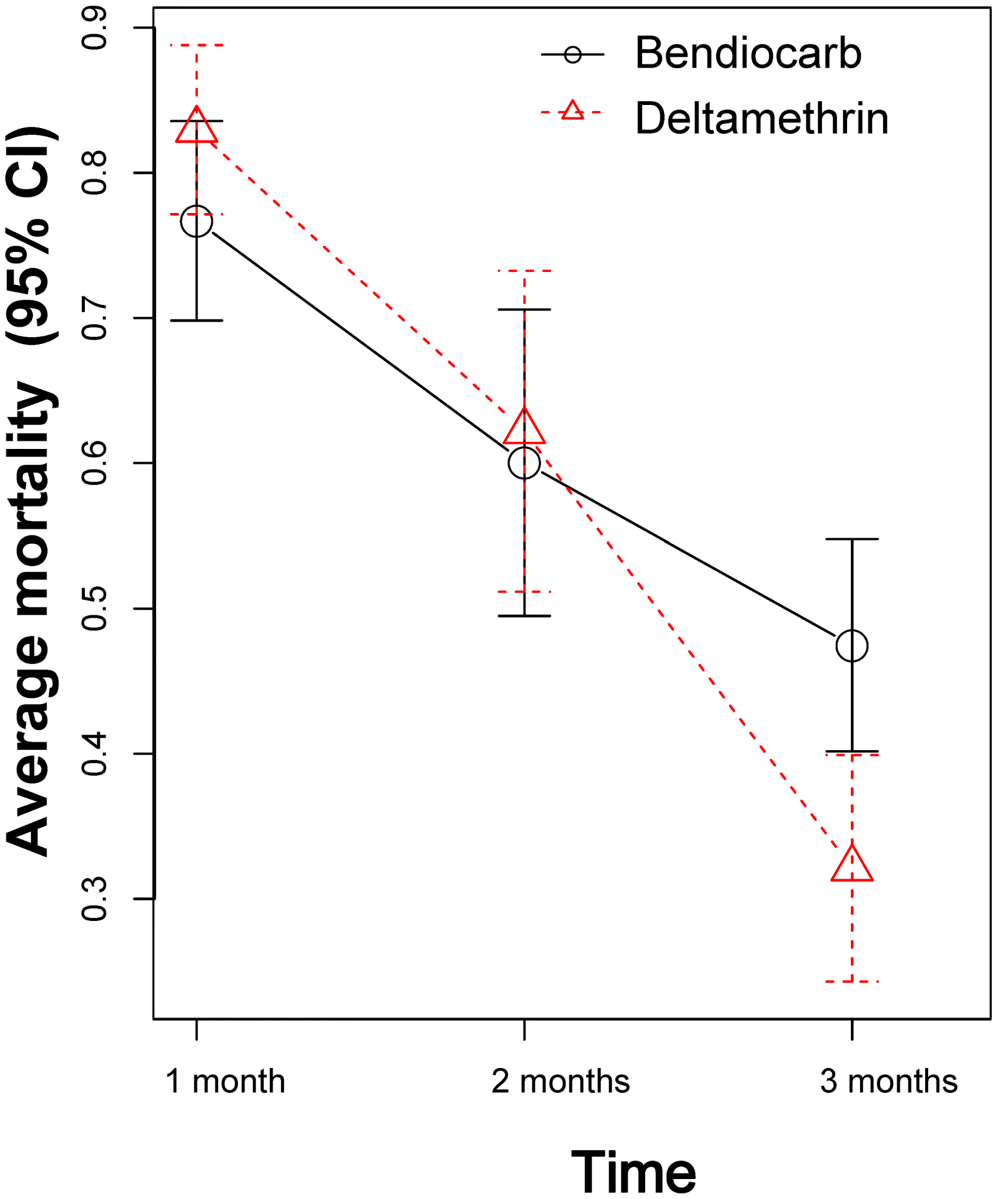
Cone bioassay data showing average mortality of susceptible *Ae*. *aegypti* (New Orleans strain) to both insecticides applied in houses belonging to this study at 1–3 months post intervention. Error bars indicate 95% CI of the mean value.

At baseline, deltamethrin susceptibility was very low, with knock-down frequencies averaging 30.7% (SD = 24.3%) at the diagnostic dose, 34.3% (SD = 23.7%) at double the diagnostic dose, 53.3% (SD = 33.9%) at five times the diagnostic dose and 73.1% (SD = 31.3%) at ten times the diagnostic dose, indicating high levels of resistance. Although deltamethrin resistance was prevalent across all sites and clusters, the neighborhood Itzincab presented the lowest levels of susceptibility for all doses tested (Fig 4). For bendiocarb, all bioassays resulted in 100% mortality at the diagnostic dose. The prevalence of both kdr mutations was very high, with 1534C found in 98% and 1016I in 71% of tested *Ae*. *aegypti* (n = 104). Cone bioassays performed in wall surfaces of 26 houses (13 Bendiocarb and 13 deltamethrin) using susceptible mosquitoes (New Orleans strain) showed 100% mortality for both insecticides at 15 days post intervention. At one month post-spraying, mortality was reduced to 78% and 83% for bendiocarb and deltamethrin and both insecticides reached average mortalities of 49% and 32%, respectively, at 3 months post intervention (Fig 5).

## Discussion

These results provide quantitative evidence suggesting that insecticide-based interventions may fail when resistance of local *Ae*. *aegypti* populations is not taken into consideration. Within an area of high resistance to deltamethrin, performing high-quality residual insecticide applications of deltamethrin indoors had no entomological impact in comparison to the application of an insecticide to which *Ae*. *aegypti* were susceptible. Deltamethrin did not produce any detectable impact against resistant *Ae*. *aepypti* during a 3-month period, whereas the application of bendiocarb was 60% more effective during a 3-month period.

Residual spraying (in the form of peri-focal spraying) was a pivotal component of the successful *Ae*. *aegypti* elimination campaign of the 1950s and 60s [35]. The dismantling of the public health infrastructure once the elimination campaign ended in the early 1970’s [1] led to the abandonment of peri-focal spraying for urban mosquito control due to difficulties in maintaining high insecticide coverage in rapidly growing urban areas. Ultra-low volume spraying (primarily truck mounted) and thermal fogging were adopted as an appealing approach for urban *Ae*. *aegypti* control due to their increased coverage, ease and speed of application, low cost and visibility to communities in comparison to the laborious peri-focal application [8]. Unfortunately, such area-wide approaches have no residual efficacy and have proven largely ineffective in preventing dengue transmission [8, 36]. Furthermore, there is indirect evidence suggesting that ULV applications are associated with rapid evolution of insecticide resistance (primarily to pyrethroids) [36, 37]. IRS is now re-emerging as an alternative *Ae*. *aegypti* control paradigm in part due to the development of new insecticide molecules (or the re-formulation of existing ones) with high potential for the control of resistant mosquitoes. The findings from this study provide initial evidence for the use of residual insecticide applications to control pyretrhoid-resistant *Ae*. *aegypti* populations, with significant reductions in population numbers up to 3 months post-spraying.

While bendiocarb IRS was effective at controlling *Ae*. *aegypti*, the method is time-consuming (it takes approximately 30–40 minutes per house) and requires strong community acceptance. Given that the “classic” form of IRS was developed for controlling malaria vectors in rural areas [26], there is room for optimizing the technique to make it more targeted to the control of *Ae*. *aegypti*. In Acapulco, Mexico, most (82%) indoor adult *Ae*. *aegypti* are found resting below 1.5m [27], leading to the possibility of focusing insecticide applications on lower resting sites as a way to reduce spraying time and insecticide costs and improve community acceptance. In Cairns, Australia, this form of targeted IRS (TIRS) led to a ~70% reduction in gravid female *Ae*. *aegypti* abundance [38] and a 86–96% reduction in symptomatic dengue cases [16]. Information about the settings which TIRS may be most effective and the scalability of this approach will be crucial components for future evaluation, including the possibility for the implementation of pre-transmission season preventive TIRS in areas identified as high-risk (e.g., schools, neighborhoods with historically high dengue transmission, etc.) or transmission hot-spots [39]. In addition, the existence of novel IRS products being brought to market for resistance management in malaria vector control could also prove to be important tools for the control of *Ae*. *aegypti*.

A key limitation of this study was the inability to age-grade collected *Ae*. *aegypti* females. As the intervention did not target peridomestic breeding sites, it is very likely that many of the adult *Ae*. *aegypti* collected indoors were recently emerged males and females which had not yet contacted a treated surface. Thus, quantifying the changes in the age structure of the adult population throughout the intervention would have helped refine our estimates of efficacy. Near infrared spectroscopy [40] constitutes a promising tool that could aid in quickly age grading of large numbers of mosquitoes as the methodology becomes increasingly more robust. An additional limitation was the limited follow up period. A longer follow up period would have allowed for a determination of the longevity of the residual effect of the insecticide, as well as how residual efficacy is related to *Ae*. *aegypti* abundance and changes in the insecticide resistance status over time.

Measuring the intensity of resistance in local *Ae*. *aegypti* populations should be considered as an important factor informing the choice of insecticide classes to be applied, as resistance intensity is considered an important correlate of vector control failure [41]. However, an operationally significant resistance threshold for *Ae*. *aegypti* has not yet been defined. In the present study, a significant proportion of the *Ae*. *aegypti* remained resistant to deltamethrin at 5 and 10 times the diagnostic dose, and no impact of the vector control intervention was detected. Given that *Ae*. *aegypti* resistance profiles appear to be highly variable in space and time, even within sub-national political units [12, 18, 19], there is a need to establish comprehensive insecticide resistance monitoring plans that can help guide public health policy. Online platforms for assembling such data have been established for *Anopheles* spp. mosquitoes [42], and are being adapted for *Ae*. *aegypti*. Such tools, combined with proper spatial analytics, can provide important information for decision makers regarding the management of insecticide resistance in *Ae*. *aegypti* and the appropriate selection of vector control tools.
